# An Integrated Monitoring Protocol to Study the Effects of Management on the C Sequestration Potential of Mediterranean Pine Ecosystems

**DOI:** 10.3390/mps9010018

**Published:** 2026-01-26

**Authors:** Nikoleta Eleftheriadou, Efstathia D. Mantzari, Natasa Kiorapostolou, Christodoulos I. Sazeides, Georgios Xanthopoulos, Nikos Markos, Gavriil Spyroglou, Evdoxia Bintsi-Frantzi, Alexandros Gouvas, Panayiotis G. Dimitrakopoulos, Mariangela N. Fotelli, Kalliopi Radoglou, Nikolaos M. Fyllas

**Affiliations:** 1 Department of Forestry and Management of Environment and Natural Resources, Democritus University of Thrace, 68200 Orestiada, Greece; alex.gkouvas@gmail.com (A.G.); kradoglo@fmenr.duth.gr (K.R.); 2 Forest Research Institute, Hellenic Agricultural Organization Dimitra, 57006 Thessaloniki, Greece; nkiorapostolou@elgo.gr (N.K.); giorgosxanth@hotmail.com (G.X.); nmarkos@elgo.gr (N.M.); spyroglou@elgo.gr (G.S.); fotelli@elgo.gr (M.N.F.); 3 Biodiversity Conservation Laboratory, Department of Environment, University of the Aegean, 81132 Mytilene, Greece; envd21005@env.aegean.gr (E.D.M.); sazeides@env.aegean.gr (C.I.S.); envd21004@env.aegean.gr (E.B.-F.); pdimi@aegean.gr (P.G.D.); 4 Department of Biology, National and Kapodistrian University of Athens, 15701 Athens, Greece; nfyllas@biol.uoa.gr

**Keywords:** *Pinus halepensis*, *Pinus brutia*, adaptive forest management, forest monitoring, forests’ dynamics, climate change

## Abstract

This article describes a field- and laboratory-based framework that can be used to monitor the C balance in Mediterranean pine forest ecosystems under different management practices that determine their structure and function. By jointly monitoring stand structure, gas exchange, litter, and decomposition dynamics, this protocol enables the assessment of how management-driven changes regulate carbon uptake, turnover, and losses, thereby affecting carbon sequestration potential. As an example, we suggest the implementation of the protocol at ten (10) permanent monitoring plots across three study areas located in Greece. The first group of plots represents a post-fire chronosequence in pine stands with no management interventions. The second group includes pine stands that exhibit variation in overstory and understory density driven by differences in microclimate and management history. The third group consists of peri-urban pine stands subjected to thinning of varying intensity. The monitoring protocol is implemented across all plots and the collected data can be classified into three analytical domains: (a) demography, encompassing measurements of tree growth and mortality; (b) litter and decomposition dynamics, involving the quantification of litterfall and its seasonality and the estimation of its decomposition rates; and (c) gas exchange, focusing on measurements of leaf photosynthesis and respiration (including relevant leaf functional traits) and monitoring of soil respiration. These three data domains can be used to comparatively consider the effect of forest management on key ecosystem processes and to constrain local-scale vegetation dynamics models.

## 1. Introduction

Human activities linked to GHG emissions, particularly CO_2_, drive global warming [1], resulting in alterations of the climate system that influence temperature and precipitation patterns [2]. Forests play a crucial role in mitigating these effects by capturing and storing carbon through photosynthesis [3] at the global scale and regulating H_2_O exchange at the regional scale. Mediterranean pine forests are under diverse management practices [4], have site-specific disturbance histories [5], and comprise plant species of distinct ecological strategies [6], forming a complex system of human–ecosystem interactions. Evaluation of adapted forest management requires robust, long-term, and comparable datasets to identify the optimum practices for C sequestration, water regulation, and ecosystem resilience under global change conditions.

In forest ecosystems, C is mainly stored in living and dead biomass as well as in the soil [7]. At the same time, C is released into the atmosphere through plant autotrophic respiration [8] and the decomposition of organic tissues [9]. Net carbon storage per unit area and time, known as Net Ecosystem Production (NEP), is defined as the difference between GPP (total photosynthetic CO_2_ uptake) and total ecosystem respiration (Reco), which includes both autotrophic (Ra) and heterotrophic (Rh) respiration [10]. An ecosystem’s role as a carbon sink or source depends on the temporal and spatial balance between GPP and Reco [11]. In Mediterranean forests, where high annual climatic variability, hot, dry summers, and irregular precipitation are observed, understanding the C balance under current and future climate change conditions is important. Photosynthetic activity, both in terms of its rate and its temporal variability throughout the year, is widely recognized as the main driver of GPP. On the other hand, Rh is mainly driven by soil water availability during the growing season and soil temperature during the colder part of the year, while Ra is mainly controlled by the dynamics of photosynthesis [8]. The way different management practices are used (such as extended rotation lengths, thinning and stand density management, understory and competing vegetation control, partial or retention harvesting, and regeneration and reforestation practices) to mediate the net carbon exchange between forests and the atmosphere is considered a nature-based climate solution [12]. In the Mediterranean region, low-elevation pine forests are either unmanaged or subjected to thinning of the overstory and partial removal of the understory, depending on local priorities. Thus, a clear understanding of their C sequestration potential based on standardized monitoring protocols [13,14], and data–model integration is needed to effectively design and evaluate climate change mitigation policies [15].

The observed and expected influence of climate change on forest productivity varies significantly by region [16]. In general, warmer conditions could have a positive effect on forest productivity in regions where water availability is not a limiting factor [17]. In the Mediterranean, where a significant temperature increase and precipitation decrease is expected by 2100, the growth of Mediterranean pines could be inhibited as the favorable effects of warmer conditions are outweighed by increased summer drought [2,18,19,20,21]. At the same time, decomposition and soil respiration in Mediterranean forests are regulated by the microenvironmental conditions and the dynamics of soil microbiota [22,23,24,25], with stand management further affecting overstory and understory biomass, litter inputs, organic matter, and soil organic carbon pools, as well as water, carbon, and nutrient cycling, and thereby influencing both carbon inputs and respiratory losses [26,27,28,29,30,31]. Monitoring of key ecosystem variables such as leaf area index (LAI) [32,33,34], photosynthesis and primary productivity [35,36,37,38], litterfall [39,40,41,42,43,44], litter decomposition [26,45,46,47,48], and soil respiration [49,50,51,52] could thus provide measurable indicators of the way different management practices control the carbon sequestration potential of a forest, by regulating its structure and function. For example, stand structure and LAI determine the stand capacity for light interception and growth, aiding the quantification of the potential effects of management on a stand’s primary productivity and biomass accumulation. In general, structural complexity is associated with higher sequestration rates [53,54,55,56,57,58]. Tree-level photosynthesis and stand-level primary productivity as regulated by stand structure could also reveal the effects of management on the carbon uptake [56,59,60,61]. Litterfall measurements track the mechanism by which canopy production returns to the forest floor, with reductions in crown density decreasing litter inputs for decomposition and associated heterotrophic respiration, allowing for the detection of management-induced changes in soil carbon dynamics and the formation of long-term soil carbon [62,63,64,65]. Soil respiration, capturing the combined influence of management on autotrophic and heterotrophic activity, provides a direct measure of how management interventions affect soil carbon losses, with a short-term increase in soil respiration after thinning and variable long-term effects depending on the recovery speed of litter inputs [62,66,67]. Taking the above into account, climate-smart adaptive forest management could be a powerful tool to increase the resilience of Mediterranean pine forest ecosystems under global change conditions and to enhance their climate change mitigation potential by means of carbon sequestration.

The aim of this study is to develop and present a field- and laboratory-based protocol for assessing the C balance of low-elevation Mediterranean coniferous forests (for example, *Pinus halepensis* Mill. and *Pinus brutia* Ten.). On the one hand, the emerging datasets can be used to comparatively consider the effect of different dominant species, microclimatic conditions, and management practices on C fluxes and to constrain local-scale forest dynamics models on the other, enabling an informed evaluation of the way different forest management practices can be used to optimize C sequestration.

## 2. Monitoring Protocol

The suggested framework integrates tree, understory, and stand-level biometric measurements, gas exchange measurements (photosynthesis and soil respiration), and litterfall and litter decomposition monitoring protocols, as well as the quantification of litter and leaf water status, aiming to provide a reproducible methodology for long-term monitoring of different low-elevation Mediterranean pine forests and management regimes.

### 2.1. Experimental/Monitoring Stages

**Figure 1:**
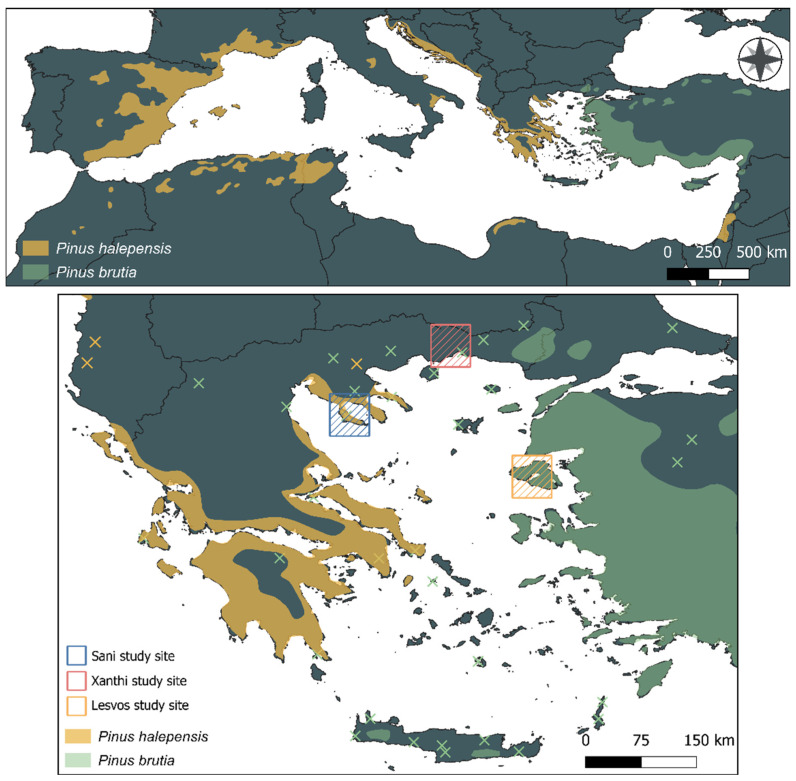
Location of the example study sites and monitoring plots in northern Greece at Xanthi, Sani, and Lesvos. The regional geographic distribution of the two pine species is also presented. The symbol “×” marks out-of-range occurrences. Distribution data is derived from Caudullo et al. [76].

1. Site selection and plot establishment: Define representative forest stands based on species composition, management history, and disturbance regime to capture contrasts in management intensity. Establish monitoring plots representing the range of within-site microenvironmental conditions. Consider including previously established monitoring plots for historic/baseline data availability. In the example study sites (see next subsection), 10 existing 0.1 ha plots were included in a multi-region, multi-plot network (Table 1, Figure 1).
2. Tree-level and understory vegetation measurements: For the tree-level measurements, mark (with a numbered label) and measure all trees with a diameter at breast height (dbh) ≥ 5 cm to track diameter, height, and crown area over time increments, as well as the tree-level survivorship. The essential measurements include the dbh (cm), the total height (m), the crown area (m^2^), and the status (dead or alive, see Section 3.1.1) of each tree in the plot. Additional measurements could include crown base height. Quantify understory biomass using field measurements of plant height and ground cover, combined with available allometric equations. An empirical allometric equation for shrub species is suggested in this protocol. Tree- and understory-level measurements quantify management-driven changes in biomass and carbon stocks.
3. Leaf area index estimation: There are different methods to estimate the leaf area index of a forest stand (LAI—m^2^ m^−2^). We suggest the ceptometer and the fisheye photographic method. Using a ceptometer, measure PAR along systematic transects to estimate LAI for both overstory and understory vegetation. Alternatively, take hemispherical photographs using a camera with a fisheye adaptor and analyze the pictures in an appropriate software to estimate LAI. Seasonal LAI measurements are recommended, particularly for stands with a significant canopy contribution from deciduous species. LAI is used as a key indicator of stand structure, reflecting forest management interventions.
4. Litterfall and litter biomass: Collect litterfall and litter at regular intervals (e.g., every two or three months) to quantify the temporal variation in organic matter pools, to distinguish between litter inputs (litterfall) and standing litter stocks (forest floor) and determine the potential biomass input for decomposition. Estimate the litter moisture content by weighing fresh and dried samples.
5. Decomposition experiment: Use site-specific pine needle litter and mixed litter in litterbags to quantify the local decomposition and, thereby, the turnover rates. Monitor microclimatic conditions such as air temperature (T_air_-°C), air relative humidity (RH-%), soil temperature (T_soil_-°C), and soil water content (SWC-%) throughout the decomposition experiment.
6. Measurements of photosynthesis and relevant functional traits: Develop light (A–I) and CO_2_ (A–Ci) response curves for needles from sunlit and shaded branches using portable gas exchange systems to estimate GPP by quantifying photosynthetic capacity, stomatal limitation, and the biochemical constraints under different management scenarios. For the same needles, measure their branch water status immediately after collection by estimating their relative water content (RWC-%) and/or water potential (Ψ-MPa).
7. Soil respiration measurements: Measure total soil (Rs-μmol m^−2^ s^−1^) and heterotrophic (Rh-μmol m^−2^ s^−1^) respiration using PVC collars and automated soil respiration systems, while simultaneously recording T_soil_ and SWC to capture management effects on carbon losses and assess how differences induced by management in soil temperature and moisture are associated with variability in respiratory carbon efflux.

The minimum monitoring period should be at least one year to include all seasons, although some measurements (like tree growth) are only useful when performed at much longer intervals. An estimation of the total duration of the protocol is approximately 540 days, with seasonal or 90-day sampling intervals for the key processes (Table 1), derived from established forest monitoring protocols [68,69,70,71,72,73,74,75] and empirical field experience to capture seasonal variability in carbon-related processes.

### 2.2. Example Study Sites

The protocol was developed and implemented at three low-elevation Mediterranean pine forest sites in northern Greece—Sani (Chalkidiki), Xanthi, and Lesvos Island—representing characteristic ecosystems dominated by *Pinus halepensis* Mill. and *Pinus brutia* Ten. (Figure 1). The sites differ in stand age, management history, and understory vegetation structure, reflecting the ecological heterogeneity of Mediterranean low-elevation forests (Table 2).

### 2.3. Chemicals

- Soda lime (CaO/NaOH) and Drierite (CaSO_4_) for the measurement of photosynthesis with infrared gas analyzers.

### 2.4. Materials

- PVC Collars (φ21.5 × 11.5 cm, any equivalent non-corrosive material of similar dimensions may be used, preferably white to reflect solar radiation).
- Plastic bins (φ 40 × 50 cm) with holes at the bottom for water drainage.
- Litter traps of known dimensions for the collection of annual litterfall and a metal or plastic frame of known dimensions for forest-floor sampling.
- Litterbags (15 × 15 cm) for decomposition measurements. The upper side of each litterbag should be made of a non-degradable mesh material (e.g., plastic net or tulle) with apertures of approximately 0.1 × 0.1 cm, allowing microbial access. The lower side should be made of a finer non-degradable mesh (e.g., polyester fabric) with apertures of approximately 0.05 × 0.05 cm to prevent material loss.

### 2.5. Equipment

- Diameter measuring tape.
- Telescopic height pole (any suitable equipment used for measuring the vegetation height).
- Drone (any suitable equipment used for taking aerial pictures of the vegetation).
- Telescopic pruning shears (the size depends on tree height; usually 5–20 m) used for sampling of the sunlit and shaded branches from the upper canopy.
- Laser measurement instrument (any suitable for measuring tree height, distances, and angles).
- Ceptometer or camera with fisheye lens/adaptor (any suitable for estimating LAI).
- T_air_ and RH sensor (any suitable for measuring air temperature and air relative humidity).
- T_soil_ and SWC sensor (any suitable for measuring soil temperature and soil water content).
- Portable leaf gas exchange system (any suitable for measuring photosynthesis and stomatal conductance under controlled light and CO_2_ conditions in the leaf chamber).
- Automated soil respiration system (any suitable for measuring soil CO_2_ efflux) and its corresponding survey chamber, T_soil_ probe, and SWC sensor.
- Portable pressure chamber (any suitable for measuring Ψ).

## 3. Procedure

### 3.1. Demography

This part of the protocol aims to monitor the growth and mortality of tree species populations within the monitoring plots.

#### 3.1.1. Estimation of the Overstory Biomass

Identify all individuals with dbh greater than 5 cm within the plots at the species level, and measure their dbh, height, crown area, and status (dead or alive). Mark each individual tree to allow re-measurement and assess its survival and growth. Estimate the aboveground biomass using species-specific allometric equations developed for similar environmental conditions (for example [77]).

#### 3.1.2. Estimation of the Understory Shrub Biomass

For individuals with dbh < 5 cm, estimate the understory biomass (*W*) based on average height and ground cover. Divide each plot into four sections. In the case of circular plots, divide each plot into four quadrants [I (0–90°), II (91–180°), III (181–270°), IV (271–360°)] using a compass. In each quadrant, determine the mean height (*H_m_*) of the understory vegetation from measurements taken at three random points. Take aerial photographs above the understory vegetation and below the overstory of each plot using a drone equipped with a camera. Using image processing software, such as the free software ImageJ version 1.54g (National Institutes of Health, Bethesda, MD, USA), calculate the percentage of understory cover within each plot (*FCC_m_*). The percentage of land cover should also be calculated, where possible, using satellite images and image processing software. Estimate the understory vegetation biomass using published allometric models or empirical equations developed for shrub and subcanopy species under comparable environmental conditions. For instance, the estimation of the aboveground biomass of the understory vegetation in Mediterranean pine forests, where the dominant species are *Quercus coccifera* L. and *Pistacia lentiscus* L. can be carried out using the equation of Pasalodos-Tato et al. [78]:

(1) $$ln\left(W\right)\;\;={\;a}_{0}+a_{1}\;\times \;ln\;\left(H_{m}\right)\;+a_{2}\;\cdot ln\;\left(FCC_{Bliss}\right)\;,$$

where

- *W* is the aboveground biomass of understory broadleaf vegetation in t ha-1;
- *H_m_* is the average height of the understory in dm;
- *a*_0_ = −0.483;
- *a*_1_ = 1.347;
- *a*_2_ = 1.174;
- *FCC_Bliss_* is calculated based on the following formula:

(2) $$FCC_{Bliss}={sin}^{-1}\left(\sqrt{\frac{FFC_{m}}{100}}\right),$$

where *FCC_m_* is the percentage of ground cover of the understory vegetation (%).

#### 3.1.3. LAI Estimation

For each plot, measure the average LAI using the ceptometer method (CMM) or through hemispherical photographs taken with a fisheye adaptor [79,80,81].


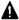
 CRITICAL STEP: Measurements should be performed at noon in six parallel lines within each monitoring plot, with a distance of approximately five meters among them, when using a ceptometer. If the hemispherical photo method is used, pictures should be taken early in the morning, just after sunrise, or under overcast conditions. A total of 36 measurements (for a plot area of around 0.1 ha) should be taken along the parallel lines throughout the plot and a measurement should be recorded every 5 m at a height of approximately 1 m from the forest floor [81]. Individual LAI measurements within a plot can be used to estimate plot-level LAI heterogeneity.

### 3.2. Litter and Decomposition Dynamics

#### 3.2.1. Litterfall and Litter Sampling

##### Litterfall

**Figure 2:**
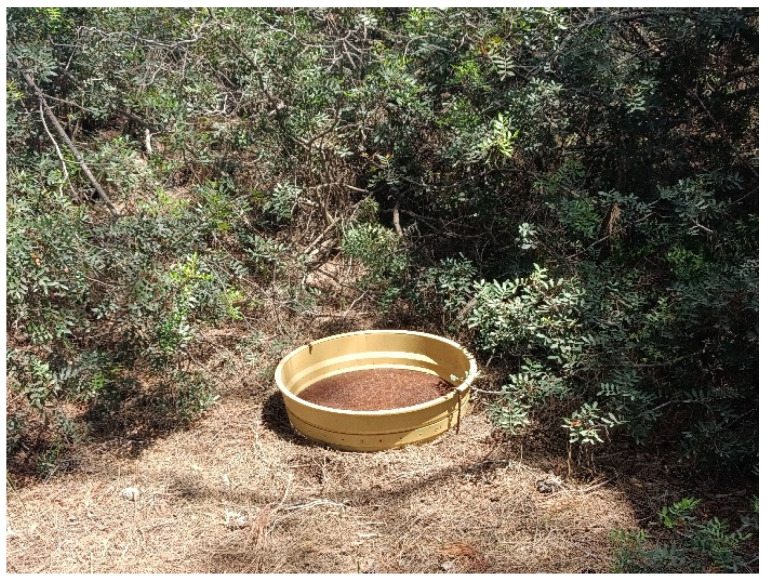
Litter trap established in a monitoring plot for the litterfall collection.

Place at least three litter traps of a known area (Figure 2) per plot to collect litterfall from both the overstory and understory of the forest stands, to quantify the dead biomass input to the forest floor. Collect the material in the litter traps inside paper bags every 90 days. Transfer the samples to the laboratory and determine their fresh weight in the field using a two-digit portable precision scale (0.01 g). In the laboratory, oven-dry the samples at 60 °C for at least 48 h until weight stabilization, allowing the determination of their dry biomass using a precision scale (0.01 g). Ideally, the litter traps should be stuck or safely installed in the ground to avoid occasional disturbance.

##### Litter

**Figure 3:**
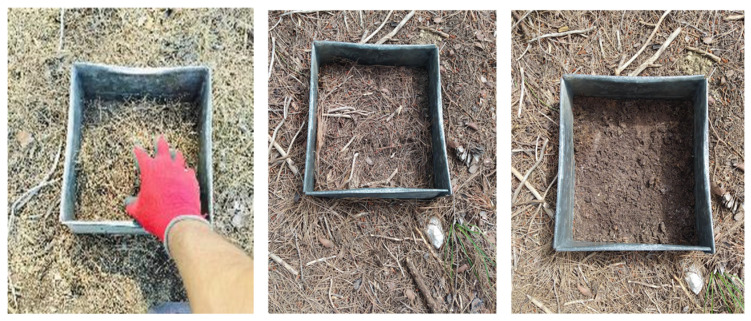
Sampling of the forest floor using a frame to collect its organic layers (L–H–F horizons).

The samplings of the forest floor should be conducted simultaneously with the litterfall sampling. Collect material from the forest floor using a frame of known dimensions, at three random points near litter traps each time.


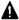
 CRITICAL STEP: Collect all the forest-floor material of the organic horizons (L, F, and H, as a single composite sample) down to—but excluding—the mineral soil (Figure 3). Avoid collecting other live herbaceous vegetation grown within the forest floor.

Place each sample in a paper bag and measure its fresh weight in the field using a portable electronic scale (measuring accuracy: 0.01 g). In the laboratory, the samples should be oven-dried at 60 °C for at least 48 h, or longer if needed, until a steady weight is achieved and their dry weight noted.

Calculate the moisture content (*WC*%) of the biomass collected from the litter traps and the forest-floor samples using the following formula:

(3) $$WC=\left(1-\frac{Dry\;mass}{Wet\;mass}\right)\times 100$$

#### 3.2.2. Decomposition Dynamics

This part of the protocol aims to investigate the dynamics of plant material decomposition in Mediterranean pine forests by estimating the decomposition rate and the abiotic and biotic factors that affect it.

To include the site-specific physicochemical factors in the decomposition process, collect fresh fallen litter from each plot (pine needles or a mixed litter sample in the case that the understory vegetation consists of a variety of species) and transfer them to the laboratory in paper bags. The collected material should be air-dried in the laboratory at room temperature for at least 48 h and subsequently placed in litterbags (15 cm × 15 cm), weighing approximately 1 g each for short total incubation periods or 2–5 g each for longer total incubation periods. Dry out an approximately 10 g sample of litter at 70 °C for at least 48 h to determine its moisture content prior to field placement. Calculate the initial dry weight of each litterbag as follows:

(4) $$W_{dry\;mass}=W_{total}-\left(W_{total}*\bar{RH}\right),$$

where *W_dry mass_* is the dry weight, *W_total_* is the wet weight, and $\bar{RH}=\frac{\bar{\Delta M}}{\bar{M_{init}}}$ is the average moisture of litter in a plot, i.e., $\bar{\Delta M}$ is the average weight loss for all plots and $\bar{M_{init}}$ is the average initial weight of the samples from all plots (~10 g).

Sequential chemical analysis of the litter is advised to monitor nutrient release during the decay process. Thus, from the initial litterfall, a sample could be collected and analyzed to find, at the least, the C/N ratio for each species contributing to the litterfall.

Individually weigh the material prior to placing it in the litterbags. The upper side of each litterbag should consist of a non-degradable organic material with mesh (e.g., plastic mesh or tulle), bearing holes approximately 0.1 cm × 0.1 cm in size to include the effect of microorganisms in the decomposition process. The underside of each litterbag should consist of approximately 0.05 cm × 0.05 cm non-degradable organic mesh material (e.g., polyester fabric) to prevent material leakage. Seal each litterbag using thread or staples.

**Figure 4:**
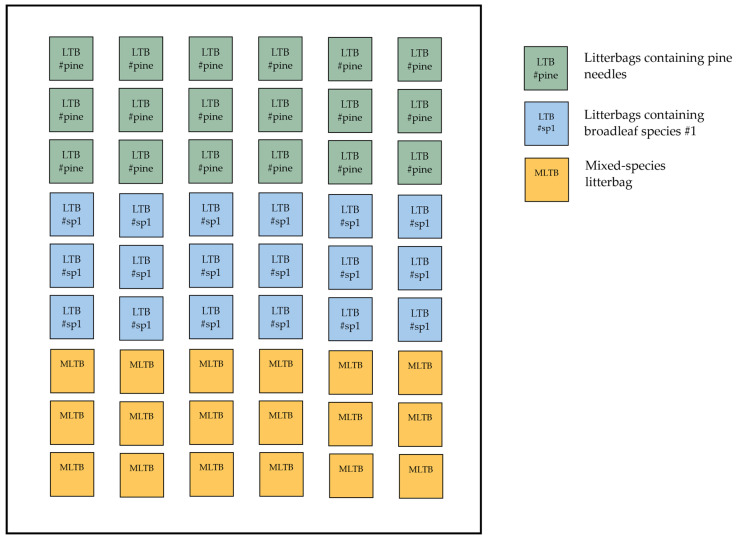
Placement of litterbags according to the decomposition protocol.

**Figure 5:**
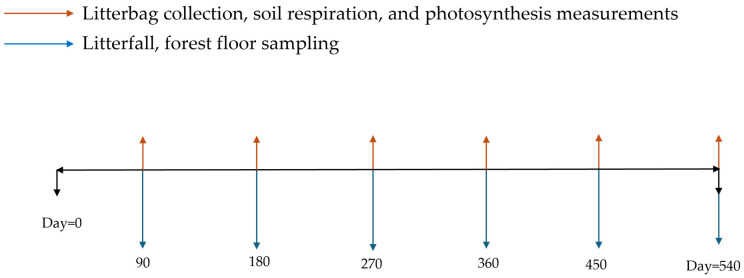
The experimental schedule for the decomposition protocol (litter and standard organic material), along with all other field assessments.

Depending on the desired monitoring period, place the equivalent number of samples. At least three samples should be collected from each treatment for each collection period as a minimum level of replication in order to include within-plot variability and allow detection of management-related differences, while maintaining feasibility for long-term monitoring. Assign a numeric label to each litterbag and place them below the forest-floor surface, above the humus layer, oriented parallel to the ground, fixed with fine wire mesh, and covered with fresh litterfall (Figure 4). Collect samples every 90 days (i.e., at days 90, 180, 270, 360, 450, and 540) (Figure 5). At each sampling event (every 90 days), carefully lift the fine mesh and collect three litterbags (or the equivalent replications) (#samples replications × #collection periods = total number of samples in each plot). Avoid disturbing the wire mesh while lifting it. After each sampling event, the litterbags should be dried for 48 h at 70 °C and the remaining mass (RM) contained should be weighed using a precision scale (0.0001 g), making sure that the before- and after-incubation weights refer to the same litterbag/sample.

In the case of mixed stands where the contribution of other species in the litter is greater than ca~10%, additional litterbags should be prepared. Estimate the percentage contribution of each species in the mixed litter (e.g., 55% pine needles, 25% species # 1, 20% species #2, etc.) per plot and prepare the litterbags using mixed litter that reflects the proportional contribution. Ideally, to assess the contribution of each species to the overall decomposition rate, set up additional sub-plots that include every species present in the stand, with enough replications to cover the entire monitoring period. Place the mixed-litter bags in the same sub-plot and collect the equivalent samples per sampling event. From each sampling, both for single-species and mixed-litter litterbags, the samples should be used to measure chemical compounds, such as the C/N ratio.

In addition, deploy the equivalent number of identical empty litterbags in both the main and supplementary decomposition sub-plots (for example, six litterbags per plot, three per sub-plot). Collect and replace these litterbags with each sampling. Use the incoming material recorded from these bags to correct for potential overestimation of the remaining mass in samples of forest-floor organic matter.

The placement of each sub-plot should be in a representative space of the plot, avoiding, if possible, vicinity to trees and high slopes. If placement occurs in plots with slope, the sub-plot should be parallel to contour lines.

**Figure 6:**
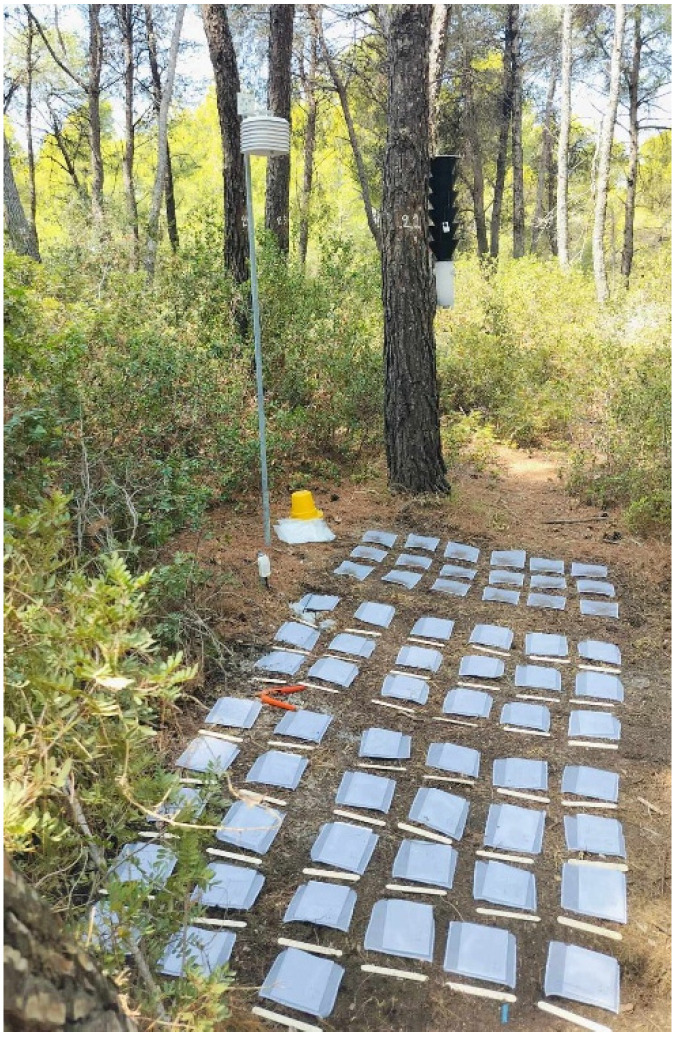
Setup of the decomposition experiment in the field and sensors monitoring the microclimatic conditions of the experiment.

On each monitoring plot, install two sensors close to where the decomposition experiment is conducted. One sensor should measure the air temperature—T_air_ and the air relative humidity—RH, and the other should measure the soil temperature—T_soil_ and the soil water content—SWC (Figure 6).

### 3.3. Gas Exchange Measurements

#### 3.3.1. Leaf Gas Exchange and Related Leaf Water Status Measurements

Perform gas exchange measurements (photosynthesis and soil respiration) once per season (for example, in January, April, July, and October) using portable instruments. Monthly measurements could provide more detailed results, but might be logistically challenging when made across many monitoring plots. The needle relative water content (RWC) and water potential (Ψ) should also be measured. Conduct all measurements on days with typical seasonal weather, based on forecasts, excluding rainy or windy conditions.

##### Photosynthetic Measurements

The purpose of these measurements is to develop response curves of the net photosynthetic rate (a) at different levels of incoming photosynthetically active radiation (PAR) (light response curves) and (b) at different CO_2_ levels (A–Ci curves) to estimate key photosynthetic traits and monitor their temporal and spatial variation.

Select and mark a minimum of five healthy, dominant, non-neighboring pine trees per plot. Given that each light response curve lasts approximately 45 min, conduct all photosynthesis measurements between 1 h after dawn and at least 2 h before sunset. In each tree, conduct measurements on needles of both a fully illuminated branch of the upper crown and on a shaded branch of the inner canopy to account for the variability in light conditions within the canopy. Carry out the seasonal measurements on the same marked individuals, unless problems arise, e.g., unavailability of accessible branches, tree health problems, etc. To obtain light response curves from the needles of cut branches, follow the methodology of Sazeides et al. [81].

Collect sunlit and shaded branches from each tree using telescopic shears (5 to 20 m, depending on tree height).


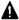
 CRITICAL STEP: Immediately place the collected branches in a bucket of water. Cut the branches again under water to prevent embolism.

Prior to placing the branch in water, a twig from each branch should be collected for relative water content (RWC) measurements (see subsequent subchapter). High-quality pruning shears should be used exclusively for this purpose, ensuring clean cuts.

**Figure 7:**
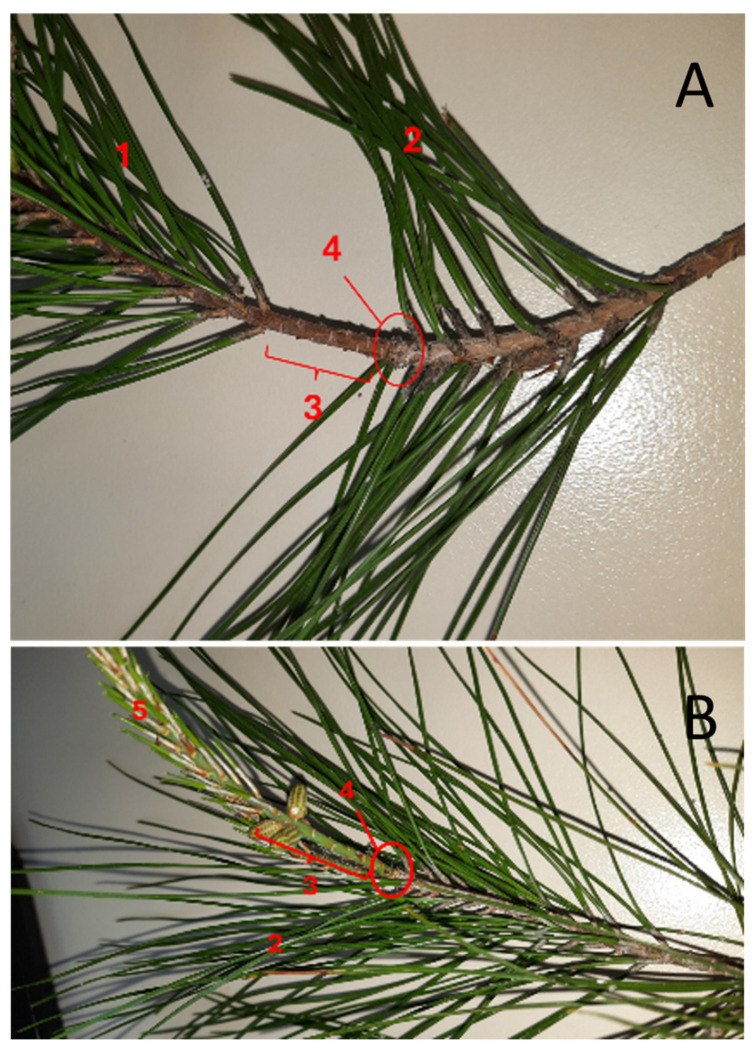
(**A**) Twig with fully developed needles of the current year. (**B**) Twig with under-developed needles of the current year. (1) Needles grown in the current year, (2) needles from the previous year, (3) the area of the branch without needles can be useful to separate sequential years, (4) an area with denser “scars” of scales, which indicates the end of the previous growing season, (5) undeveloped needles from the current year.

Perform gas exchange measurements using enough needles to cover the surface of the chamber of the portable photosynthesis system, taking care to avoid needle overlapping. If complete coverage is not possible, perform measurements on three pairs of fully developed and healthy needles from both illuminated and shaded branches. In these cases, mark the needle edges and correct the measurements for the enclosed leaf area. Perform measurements on shaded and sunlit branches alternately to ensure the following: (1) temporal proximity, allowing direct comparison between the two and (2) that each set of measurements—whether from shaded or sunlit branches—is taken at a different time during the day, thereby accounting for hourly variations in temperature and humidity. Select only needles grown in the current year (Figure 7A(1)) or needles from the previous year (Figure 7B(2)) when the needles from the current year are not fully developed (Figure 7B(5)).

For the light response curves, needles should first be acclimated under PAR of 1000 μmol m^−2^ s^−1^ for shaded needles and 1600 μmol m^−2^ s^−1^ for sunlit needles. Wait until stomatal conductance exceeds 0.02 mol m^−2^ s^−1^ and stabilizes or, alternatively, the transpiration values are above 0.11 mmol m^−2^ s^−1^ for PAR at 1000 μmol m^−2^ s^−1^, or 0.12 mmol m^−2^ s^−1^ for PAR at 1600 μmol m^−2^ s^−1^. Set the chamber (block) temperature equal to the atmospheric temperature at the start of the measurement. Generate the light response curves by setting the chamber humidity and temperature at ambient levels, the stomatal ratio to 0.5, the CO_2_ flow rate at 500 (µmol s^−1^), the CO_2_ reference concentration at 420 ppm, and the consecutive PAR steps at 2000, 1800, 1600, 1400, 1200, 1000, 800, 600, 400, 200, 100, 80, 60, 40, 20, and 0 μmol m^−2^ s^−1^ to measure the net photosynthesis, A_net_ (μmol m^−2^ s ^−1^).


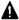
 CRITICAL STEP: Under humid ambient conditions, keep the RH of the chamber at a level up to 70%. This is very critical, especially on days with high temperatures, to avoid contamination.

At each PAR level, three A_net_ logs should be taken after at least two minutes, allowing the needles to reach equilibrium. Dark respiration (Rd) can be estimated based on the parameters of the photosynthesis light response curve [constant term (intercept) for PAR = 0].

In addition to the light response curves, photosynthesis–CO_2_ (A–C_i_) curves are useful for model parameterization and the quantification of additional photosynthetic parameters. Using a branch from a sunlit part of the crown, place the needles in the gasket as above and set the block temperature at 25 °C and the incoming PAR at 1800 μmol m^−2^ s^−1^. Keep these settings constant throughout the measurements. Set the CO_2_ concentration sequence as follows: 400, 300, 200, 150, 125, 100, 75, 50, 40, 400, 400, 600, 900, 1250, 1500, 1750, and 2000 ppm and perform five measurements for each level.

After finalizing all gas exchange measurements, cut down the three pairs of needles used and transport them to the laboratory, where they should be stored in moist and dark conditions (e.g., in a refrigerator in a sealed plastic bag). On the same or the following day, scan the needles, and calculate the leaf area corresponding to the section within the chamber using image analysis software, such as ImageJ [82].

##### Branch and Needle Relative Water Content Measurements

**Figure 8:**
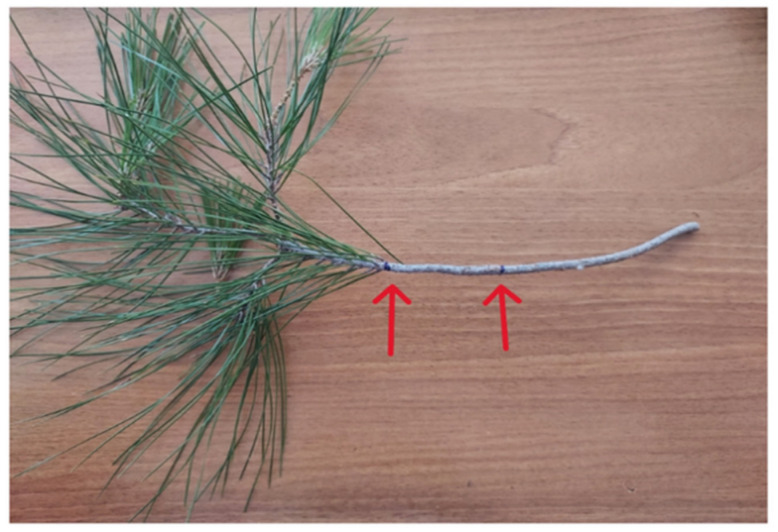
Indicative twig cutting points for relative water content measurements.

For the measurements of relative water content (*RWC*), use one twig from each of the branches collected for the photosynthesis measurements. From each branch, collect a twig immediately after the branch is cut. From each twig, collect one twig sample (10 cm in length, measured from the base of the needles, see Figure 8) and four needle samples, each consisting of a fascicle (pair) of needles.

If the monitoring plots are remote, use a portable electronic scale (accuracy of 0.001 g) to weigh the twig and needle samples and put each of them in pre-labeled Falcon-type tubes containing distilled water. Store the tubes in a coolbox for 24 h or until transferred to the laboratory.

When possible, it is better to determine the fresh weight of the samples in the laboratory on the day of sampling. In this case, pre-weighted and labeled Falcon-type tubes should be used in the field to contain each sample. Then, weigh the samples in the tubes at the laboratory using a high-precision scale (0.0001 g) for greater accuracy, add distilled water, and store the tubes in the refrigerator for 24 h.

After the water saturation period, for the estimation of the needle and twig turgid weight (*TW*), place each sample on absorbent paper in the laboratory to remove excess moisture and re-weigh it. Then, place the samples in an oven at 80 °C for 24 h and re-weigh them to estimate their dry weight (*DW*).

Calculate the *RWC* of the needle and twig samples as follows:

(5) $$RWC\;\left(\%\right)=\;\frac{W-DW}{TW-DW}*100$$

For each twig sample, record the diameter, measured at the middle of each branch, after the measurements are completed.

##### Leaf Water Potential (Ψ) Measurements

For the leaf Ψ assessment, perform measurements with a portable pressure chamber, especially during the dry summer period. Perform measurements both before dawn (predawn) and at midday (between 12:00 and 15:00), on needles collected from the same trees selected for photosynthesis measurements.

From each tree, collect a small twig, cover the needles with aluminum foil, and place it inside a pre-marked polybag to avoid desiccation prior to measurement. Insert the petiole through the bottom of the cap, secure the petiole with an appropriately sized rubber seal, place the cap onto the chamber, and cleanly cut the end of the petiole outside the chamber. Once the cap with the sample is secured, initiate pressurization. Observe the exposed end of the petiole until the first appearance of sap and note the pressure (MPa) at which water appears, corresponding to the leaf water potential.


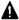
 CRITICAL STEP: In the case of pines, the first liquid to appear is usually resin, so caution is advised to distinguish xylem sap from resin. Resin is characterized by higher viscosity, while xylem sap emerges in small, distinct bubbles under the resin layer created by pressure. For safety reasons, it is highly recommended to use safety glasses when performing the measurements.

#### 3.3.2. Soil Respiration Measurements

**Figure 9:**
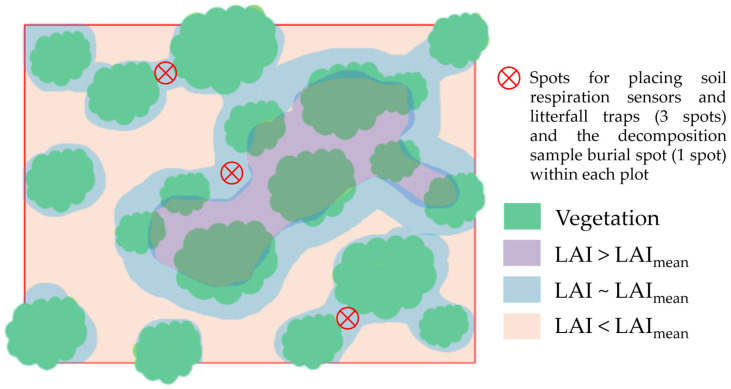
Location of soil respiration monitoring spots within each plot.

Three months prior to the first measurement, install at least four PVC collars (21.5 cm in diameter and 11.5 cm in height, depending on the guidelines of each soil respiration system) in each monitoring plot to assess soil CO_2_ efflux. Select spots with an LAI that is representative of the plot’s LAI and establish the collars randomly at such spots within the plot (Figure 9).

**Figure 10:**
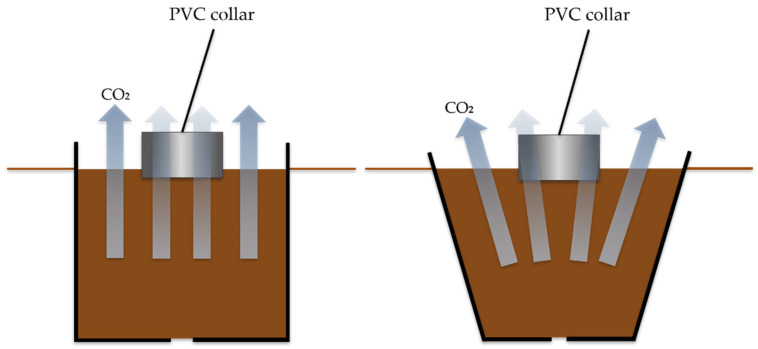
Differentiation of CO_2_ diffusion by the shape of the bin.

Insert one collar per plot at 5 cm into the soil to measure Rs, and use the remaining three collars for Rh. For Rh, excavate three trenches (approximately 50 cm in diameter and 70 cm deep) in each plot, and insert plastic bins (40 cm diameter × 50 cm height) bearing drainage holes (Figure 10). Refill these bins in layers with root-free soil to reproduce natural soil structure (following [83,84]) and position a PVC collar at 5 cm depth within each bin. To ensure consistent CO_2_ flux measurements and prevent errors/biases due to diffusion differences, the same bin shape and dimensions should be used in all plots [85] (Figure 10).


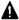
 CRITICAL STEP: One day before or immediately prior to the measurements, carefully remove any green or live vegetation within the collars to avoid interference. Caution should be taken to avoid disturbing the litter inside the soil collar. For all soil CO_2_ efflux measurements, the following parameters should be standardized in the corresponding software of the instrument to ensure consistency [86]:

- Observation length: 120 s;
- Observation count: 3;
- Pre-purge: 30 s;
- Post-purge: 15 s;
- Dead band: 15 s;
- Chamber offset: The offset of each collar.

In addition, record T_soil_ and SWC with sensors connected to the soil respiration system. During soil respiration assessments, insert both sensors directly into the root-free soil inside the plastic bins (for Rh) or in the soil close to the collar (for Rs), to ensure accurate readings of abiotic soil conditions.

Measurements should be made seasonally, between 09:00 and 17:00 (or adjusted accordingly depending on the season) to capture daytime variation in CO_2_ flux. For each sampling day, complete three replicates during morning, midday, and afternoon periods.


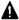
 CRITICAL STEP: Soil CO_2_ efflux measurements should not be conducted during or immediately after rainfall, as surface water alters gas diffusion, T_soil_, and SWC, leading to unreliable values.

## 4. Expected Results

**Figure 11:**
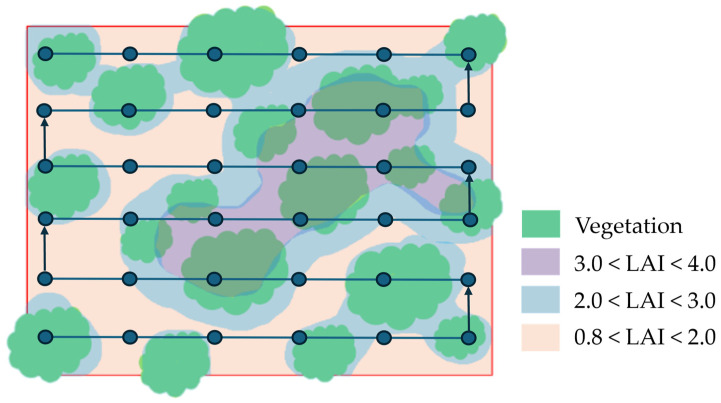
Overview of the plot showing the layout of transects used for the leaf area index (LAI) measurements and the corresponding expected LAI levels across the area.

Variation in the LAI is expected to reflect the structural heterogeneity among sites, driven by differences in stand age and forest management. Stands characterized by higher canopy density (both overstory and understory) are anticipated to exhibit higher LAI values, in contrast to stands with a lower canopy density (Figure 11). Although LAI is not the only variable expressing stand structure, its use is frequent for both field and modeling studies [32,33,34]. Overstory LAI values can be broadly classified into three density categories [87,88]:

- Low density (LAI ranging approx. from 0.8 to 2.0 m^2^/m^2^)
- Medium density (LAI ranging approx. from 2.0 to 3.0 m^2^/m^2^)
- High density (LAI ranging approx. from 3.0 up to 4.0 m^2^/m^2^)

Forest management influences stand structure, particularly canopy density and microclimatic conditions, through practices such as thinning of the overstory or removal of the understory. LAI is a key indicator of stand structure, frequently used in ecological and forest models. Given that management practices, such as thinning, directly influence LAI values in managed vs. unmanaged stands [32,89,90,91], LAI can be used to reflect forest management interventions. Due to the strong dependency of photosynthesis on light availability, structural changes induced by management (e.g., opening of the canopy) are expected to alter the light availability of each tree within the stand. Thus, thinning is expected to increase light availability to previously shaded individuals [35], potentially increasing their photosynthetic rates [36,37,38], and, consequently, GPP, which may enhance C uptake at the stand level if the photosynthesis increment is not counterbalanced by higher respiratory C losses [92]. Conversely, in denser stands, whether this is due to younger stand age or the absence of management, the foliage of smaller trees is expected to face more shading and achieve reduced photosynthetic rates [93]. This phenomenon is also reflected within a single tree canopy, with shaded needles demonstrating lower photosynthetic rates compared to sunlit ones [94].

**Figure 12:**
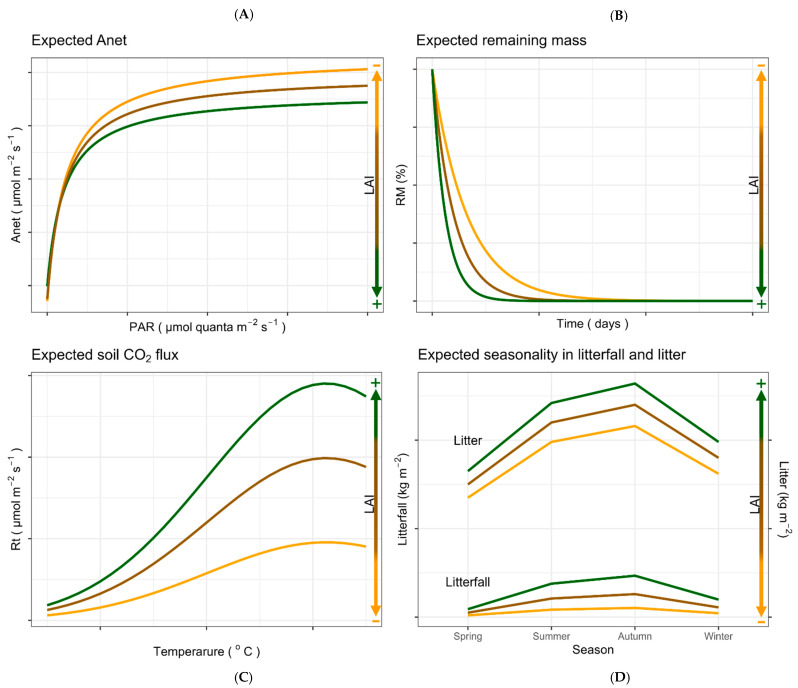
Expected responses of (**A**) net photosynthesis (Aₙₑₜ), (**B**) remaining litter mass (RM), (**C**) soil CO_2_ efflux, and (**D**) litterfall and forest-floor litter biomass to variation in leaf area index (LAI).

During a typical light response curve, it is expected to observe a sharp rise at low PAR (0–200 µmol m^−2^ s^−1^), followed by a slower increase at medium PAR (200–800 µmol m^−2^ s^−1^), and a plateau when PAR reaches 800–2000 µmol m^−2^ s^−1^, near A_max_. Photoinhibition of photosynthesis may also be observed at the highest PAR levels. A higher A_max_ and saturation at the highest PAR should be expected in sunlit needles compared to shaded needles [94], as well as in plots with a lower LAI compared to those with a higher LAI (Figure 12A).

Seasonal variation in soil CO_2_ efflux usually reflects both stand structure and climate variation between sites [49]. Total soil CO_2_ efflux is expected to be consistently higher than the heterotrophic component across all seasons. Seasonal variation in Rs is also expected, with higher efflux rates generally anticipated during spring and the lowest during the winter and/or summer dry period, reflecting enhanced microbial and root activity under favorable T_soil_ and SWC conditions [50,51,52]. Soil respiration rates generally increase with temperature due to higher microbial and root metabolic activity, with soil moisture regulating the availability of oxygen and substrate and setting thresholds for soil respiration rates. Increased soil dryness leads to suppression of microbial activity, while extremely wet soil conditions, on the other hand, cause oxygen limitation, resulting in both cases in reduced total soil respiration [95,96,97]. This pattern should also be expected diurnally. During the morning, Rs remains relatively low due to a cooler T_soil_ and limited a supply of photosynthetic products to the rhizosphere and soil. As T_soil_ rises and photosynthesis increases, both microbial and root respiration increase, resulting in maximum soil respiration around midday. In the afternoon, respiration decreases as soil temperature and photosynthetic activity decline, and stomatal closure limits C supply to the roots [98,99]. Management practices such as thinning and understory vegetation removal alter canopy cover, as well as the root density, litter inputs, and microclimate (soil temperature and moisture), all of which can contribute to spatial and seasonal differences in soil respiration.

Thinning is expected to increase soil respiration during the growing season by increasing soil temperature, moisture, and nitrate availability, which stimulate root growth and microbial activity. In contrast, thinning practices could lower soil temperature in the non-growing season, resulting in reduced soil respiration [21,100] (Figure 12B). Understory density also influences soil respiration. High understory density can increase soil respiration by contributing to root respiration and litter input, but can also reduce it in cases where water competition limits root activity [101]. In addition, soil respiration is expected to increase with stand age as a result of accumulated litter inputs and root biomass, while younger stands are anticipated to have comparatively lower soil respiration [102].

The soil temperature rise caused by thinning and understory vegetation removal, as well as stand age, is also expected to affect litter decomposition. Thinning generally increases decomposition rates in pine plantations by modifying litter quality and improving microclimatic conditions, accelerating nutrient cycling (N accumulation and release) [103] (Figure 12C), thereby resulting in a faster C turnover and a potentially shorter soil C storage time [104]. Thinning intensity, however, produces substrate-dependent effects. Heavy thinning may slow needle decomposition [26]. On the other hand, stand age may affect decomposition as older pine stands accumulate more litter and root biomass, providing more substrate for microbial decomposition, with decomposition generally increasing as stands mature and litter inputs accumulate [45]. Thus, it is expected that stands with a higher basal area and LAI are generally characterized by higher litter decomposition rates [46,47,48,105].

It is expected that the litterfall biomass will demonstrate seasonality and be driven by site- and species-specific factors [39]. Litterfall is expected to peak during autumn and summer, mainly due to the leaf senescence and drought-induced shedding associated with the Mediterranean climate [40], while during winter and spring, lower litterfall rates are expected. Forest-floor biomass is generally anticipated to follow the seasonal pattern of the litterfall biomass. Forest-floor biomass tends to increase following periods of intense litterfall and when cooler conditions in the winter and drier conditions in the summer favor the retention of organic material on the soil surface [41]. With the onset of spring, higher temperatures and soil moisture stimulate microbial and faunal activity, enhancing C and N cycling and leading to a decline in accumulated litter [42]. Beyond seasonal variation, management interventions also play a significant role in regulating litterfall and forest-floor biomass. Thinning and understory vegetation removal reduce stand canopy density and lead to lower litter inputs and decreased accumulation on the forest floor [43], although effects might vary with thinning intensity [44]. Therefore, stands with higher LAI are expected to demonstrate higher litterfall and litter accumulation compared to those with lower LAI (Figure 12D), reflecting a greater canopy productivity and enhancing soil C inputs [106]. However, the net C effect is closely related to the decomposition rates and soil CO_2_ efflux, which are also influenced by management [64].

Data from the implementation of the proposed monitoring protocol could be used to comparatively evaluate the effect of different management practices on the C sequestration potential of Mediterranean pine forests. The evaluation could be pursued following a purely empirical/statistical perspective and/or a mechanistic understanding based on process-based simulations. Statistical analysis of such datasets could use the framework of mixed-effect modeling [107], using the “Plots” as random effect terms to account for the hierarchical structure of the dataset and the various environmental predictors as fixed effect terms. For example, modeling the observed litter decomposition or soil respiration rate as a function of the prevailing environmental conditions and allowing for a varying plot effect could yield a generic equation for describing how these rates are affected by climate and/or stand-level variables, and whether between-plot variation is significant or not. Such types of equations could alternatively be used to parameterize local-scale process-based models that simulate C fluxes at the stand or tree level [108,109,110]. Local-scale parameterization and incorporation of functional trait plasticity that is related to C sequestration could provide more realistic simulations of forest productivity under current and future climate change conditions [111] and explore the role of different interventions in adaptive forest management [13,112].

## 5. Conclusions

Management practices that alter forest stand density affect carbon sequestration structurally and functionally. Light availability, photosynthetic capacity, litter inputs, soil microclimate, and carbon losses through respiration are all affected by management practices. Across sites and management intensities, integrating these components allows for the consistent evaluation of the management effects on carbon uptake, turnover, and storage, thereby framing the protocol as an integrated mechanism for monitoring forest carbon dynamics.

Many previous studies have examined the individual processes. For instance, some focused on soil respiration responses to management [62,66], litter decomposition dynamics [113], or stand biomass and carbon stocks in forest pools [114,115], or have examined single experimental sites within Mediterranean regions [114,115,116]. This protocol provides a harmonized framework that monitors stand structure (LAI and biomass), carbon inputs (photosynthesis and litterfall), and carbon losses (soil respiration and decomposition), facilitating the detection and interpretation of management-induced changes in the carbon balance of the ecosystem, while providing standardized methodologies for data collection on the key ecosystem components that regulate carbon dynamics in managed forests. Therefore, it offers a scientific basis for comparing management regimes and supporting forest management strategies, aiming at optimizing carbon sequestration under Mediterranean environmental constraints, while facilitating collaborative monitoring between researchers and forest authorities to inform management decisions.

## Figures and Tables

**Table 1:** The monitoring protocol and the time needed to complete every stage.

Process	Description	Duration	Sampling Interval
Site setup and marking	Plot establishment, establishment of sensors	1 month	—
Tree and understory biometric data	Tree and understory measurements	2–3 days per plot	Every 5 years (optimal)
LAI measurement	PAR readings via ceptometer or fisheye photos	1 day per plot	Seasonal (Jan, Apr, Jul, Oct)
Litterfall and litter sampling	Biomass and moisture assessment	540 days	Every 90, 30, or 15 days
Decomposition experiment	Litterbags and standardized materials	540 days	Every 90 days
Ecophysiological measurements	Photosynthesis, RWC	1–2 days per plot	Seasonal (Jan, Apr, Jul, Oct)
	Needle Ψ (predawn and midday)	2 days per plot	Annual (preferably once during the dry season and once before autumn precipitation begins)
Soil respiration	Total and heterotrophic soil CO_2_ efflux	1–2 days per plot	Seasonal (Jan, Apr, Jul, Oct)

**Table 2:** Summary of the expected results of different management practices on total biomass, LAI, litter, decomposition, and gas exchange across the three example study sites (Lesvos, Sani, Xanthi).

Species	Site	Management	Effect on Total Biomass	Effect on LAI	Effect on Litter and Decomposition	Effect on Gas Exchange
*P. brutia*	Lesvos	None	Increases over time following a fire, reaching a plateau	Increases over time, eventually reaching a plateau before declining slightly	Increasing litter inputs with increasing stand age, slow decomposition under dense canopy	Photosynthesis increases with canopy development, soil respiration changes depending on the soil water content
*P. halepensis*	Sani	Understory removal only	Overstory biomass remains stable; understory biomass decreases with increasing removal intensity	Overstory LAI remains relatively constant; total LAI decreases as understory removal intensity increases	Decreasing litter inputs and increasing decomposition rates with increasing understory removal	Photosynthesis remains relatively stable with understory removal, soil respiration changes depending on the soil water content
*P. brutia*	Xanthi	Thinning intensity	Overstory biomass decreases as thinning intensity increases	Decreases with increasing thinning intensity	Decreasing litter inputs and increasing decomposition rates with increasing thinning	Photosynthesis increases with thinning, soil respiration changes depending on the soil water content

## Data Availability

No new data were created or analyzed in this study. Data sharing is not applicable to this article.

## Abbreviations

The following abbreviations are used in this manuscript:

Ecosystem fluxes	
C	Carbon fluxes
H_2_O	Water fluxes
GHG	Greenhouse gases
CO_2_	Carbon dioxide
NEP	Net ecosystem productivity
GPP	Gross primary production
Demography	
dbh	Diameter at breast height (cm)
LAI	Leaf area index (m^2^ m^−2^)
W	Understory aboveground biomass (t ha^−1^)
Hm	Mean understory height (m)
FCCm	Understory ground cover (%)
Litter and decomposition dynamics	
WC	Litter moisture content (%)
W_total_	Initial litter wet mass (g)
W_dry mass_	Initial litter dry mass (g)
RM	Remaining litter mass (g)
T_air_	Air temperature (°C)
RH	Relative humidity (%)
T_soil_	Soil temperature (°C)
SWC	Soil water content (%)
Leaf gas exchange	
PAR	Photosynthetically active radiation (μmol m^−2^ s^−1^)
A_net_	Net photosynthetic rate (μmol m^−2^ s^−1^)
Rd	Dark respiration (μmol m^−2^ s^−1^)
Leaf water status	
RWC	Relative water content (%)
Ψ	Leaf water potential (MPa)
Soil respiration	
Reco	Ecosystem respiration (μmol m^−2^ s^−1^)
Ra	Autotrophic respiration (μmol m^−2^ s^−1^)
Rh	Heterotrophic respiration (μmol m^−2^ s^−1^)
